# Prediction significance of autophagy-related genes in survival probability and drug resistance in diffuse large B-cell lymphoma

**DOI:** 10.18632/aging.205282

**Published:** 2024-01-17

**Authors:** Dan Xiong, Xiaolei Wei, Weiming Huang, Jingxia Zheng, Ru Feng

**Affiliations:** 1 Department of Hematology, Nanfang Hospital, Southern Medical University or the First School of Clinical Medicine, Southern Medical University, Guangzhou 510515, China; 2 Department of Hematology, Shunde Hospital, Southern Medical University (The First People’s Hospital of Shunde), Foshan 528308, Guangdong, China

**Keywords:** diffuse large B-cell lymphoma, autophagy, prognosis, immune infiltration, drug resistance

## Abstract

Background/Aims: Diffuse large B-cell lymphoma (DLBCL), the most common subtype of non-Hodgkin lymphoma, has significant prognostic heterogeneity. This study aimed to generate a prognostic prediction model based on autophagy-related genes for DLBCL patients.

Methods: Utilizing bioinformatics techniques, we analyzed the clinical information and transcriptome data of DLBCL patients from the Gene Expression Omnibus (GEO) database. Through unsupervised clustering, we identified new autophagy-related molecular subtypes and pinpointed differentially expressed genes (DEGs) between these subtypes. Based on these DEGs, a prognostic model was constructed using Cox and Lasso regression. The effectiveness, accuracy, and clinical utility of this prognostic model were assessed using numerous independent validation cohorts, survival analyses, receiver operating characteristic (ROC) curves, multivariate Cox regression analysis, nomograms, and calibration curves. Moreover, functional analysis, immune cell infiltration, and drug sensitivity analysis were performed.

Results: DLBCL patients with different clinical characterizations (age, molecular subtypes, ECOG scores, and stages) showed different expression features of autophagy-related genes. The prediction model was constructed based on the eight autophagy-related genes (ADD3, IGFBP3, TPM1, LYZ, AFDN, DNAJC10, GLIS3, and CCDC102A). The prognostic nomogram for overall survival of DLBCL patients incorporated risk level, stage, ECOG scores, and molecular subtypes, showing excellent agreement between observed and predicted outcomes. Differences were noted in the proportions of immune cells (native B cells, Treg cells, CD8^+^ T cell, CD4^+^ memory activated T cells, gamma delta T cells, macrophages M1, and resting mast cells) between high-risk and low-risk groups. LYZ and ADD3 exhibited correlations with drug resistance to most chemotherapeutic drugs.

Conclusions: This study established a novel prognostic assessment model based on the expression profile of autophagy-related genes and clinical characteristics of DLBCL patients, explored immune infiltration and predicted drug resistance, which may guide precise and individualized immunochemotherapy regimens.

## INTRODUCTION

Diffuse large B-cell lymphoma (DLBCL) represents the most common subtype of non-Hodgkin lymphoma, characterized by diverse clinical presentations, biological attributes, and prognostic outcomes. The standard treatment of DLBCL is chemo-immunotherapy with R-CHOP (rituximab, cyclophosphamide, doxorubicin, prednisone, vindesine, and bleomycin), which leads to a significant improvement in overall survival. Although this modality is safe and effective, only about 60% of cases can be cured with the standard R-CHOP treatment, leaving around 40% to face relapses or treatment resistance [1, 2]. Patients with relapsed or refractory diffuse large B-cell lymphoma (r/r DLBCL) face a challenging prognosis. As such, risk prediction consequently remains pivotal for DLBCL intervention. Precise prognostic evaluation, particularly at the initial point of diagnosis, plays a critical role in shaping treatment strategies and enhancing the likelihood of survival. Clinicians analyzed the risk factors including age, lactate dehydrogenase, extra-nodal sites, stage, and performance status, and developed the international prognostic index (IPI) to characterize the prognosis of aggressive non-Hodgkin lymphoma [3]. Scores of 0-1, 2, 3, and 4-5 by IPI correspond to a 3-year overall survival of 91%, 81%, 65%, and 59%, respectively [4]. Besides, DLBCL subtypes and molecular features offer another dimension to identify populations with high risk, which are independent of IPI [5, 6].

Ongoing efforts profoundly advance the understanding of the genomic and transcriptomic landscape of DLBCL [7]. Several risk prediction models were developed based on the profiles of immune infiltration [8] and angiogenesis [9]. Most interestingly, it was revealed that the expression profile of autophagy-related genes can be used to predict the prognosis of DLBCL [10]. This study then explored the molecular mechanism of autophagy-related genes in the occurrence and process of DLBCL.

Autophagy facilitates metabolic adaptation and mediates nutrient cycling, and this multistep lysosomal degradation participates in the cancer process [11, 12]. The expression profiles of autophagy-related genes are widely used to establish prediction models for prognosis or overall survival in prostate cancer, breast cancer, clear-cell renal cell carcinoma, and glioma [13–16]. Furthermore, recent findings support the application potential of therapeutic molecules that target functional mechanisms of autophagy for the treatment of DLBCL [17, 18], and the results implied that autophagy-related genes can serve as prognostic markers for DLBCL. In this study, the prognostic assessment model was established based on the expression profile of autophagy-related genes and clinical features of DLBCL patients. Next, the risk scoring model was applied to predict immune infiltration and drug resistance. Our findings may provide clinical potential in therapeutic interventions for individual cases of DLBCL.

## MATERIALS AND METHODS

### Data extraction and processing

### Model building dataset

We extracted clinical and transcriptomic data for 119 DLBCL patients from the GSE53786 dataset (https://www.ncbi.nlm.nih.gov/geo/query/acc.cgi?acc=GSE53786) in the Gene Expression Omnibus (GEO) database. Specifically, we used the dataset, retrieved on 16 January 2022 using the GEOquery package (v2.60.0) [19], and analyzed with the GPL570 microarray platform, as the source for constructing our prognostic model. The baseline features of these patients, including age, subtypes, ECOG score, stage, and survival probability, are detailed in Table 1. Patient staging adhered to the American Joint Committee on Cancer tumor node metastasis (TNM) system [20].

**Table 1:** Clinicopathological characteristics among 2 molecular subtypes of DLBCL.

**Characteristics**		**Cluster 1**	**Cluster 2**	**P-values**
Age (years)	< 60	18	32	0.5192
	≥ 60	28	36	
Gender	Females	22	26	0.4098
	Males	24	42	
ECOG scores	0	8	16	0.128
	1	21	26	
	2	14	11	
	3	1	8	
	4	0	1	
Subtypes	ABC	17	29	0.3195
	GCB	17	29	
	Unclassified	12	10	
Tumor TNM stage	I	6	10	0.9162
	II	13	22	
	III	12	17	
	IV	14	17	

ABC, activated B cell-like DLBCL; GCB, germinal center B cell-like DLBCL; DLBCL, diffuse large B-cell lymphoma. The Chi-square test was used to compare categorical variables. P < 0.05 indicates statistical significances.

### Validation datasets

To further validate the predictive effect of the model, two additional datasets, GSE10846 and GSE181063, were chosen from the GEO database due to their direct relevance to DLBCL. These datasets encompass detailed clinical information, including age, disease stage, treatment approaches, and follow-up records, enabling nuanced analyses and ensuring the applicability of our model’s results to practical clinical scenarios. Furthermore, they originate from respected studies published in prestigious journals, attesting to their quality and reliability. GSE10846 is an array dataset containing gene expression patterns of 306 DLBCL patient samples, and 6 samples were excluded in this study given missing survival information. GSE181063 presents gene expression profiles of 1310 biopsies from patients diagnosed with DLBCL, and 7 clinical samples were not included in this study because of lacking survival information.

### Gene annotation and selection

Gene annotation was performed using hgu133plus2.db (v3.13.0). Autophagy-related genes were derived from the following databases: HADb (available at http://www.autophagy.lu/index.html), HAMdb (available at http://hamdb.scbdd.com/), and AUTOPHAGY DATABASE (available at http://www.tanpaku.org/autophagy/) on 18 January 2022.

### Unsupervised subtype classification

Unsupervised machine learning was performed using ConsensusClusterPlus R package (v1.36.0) [21] to identify autophagy-based molecular subclasses of DLBCL, with a maximum cluster number set to 6. The optimal cluster number was determined by evaluating the consensus matrix heatmaps and cluster-consensus values [22]. The preferred number of clusters was selected based on the criteria of achieving the clearest heatmap and highest cluster-consensus values, indicating higher stability within the clusters. The cumulative distribution function (CDF) of the consensus matrix for *κ* clusters is a more quantitative measure of cluster coherence. Then a delta area plot was produced to display the amount of change in area under the CDF between *κ* and *κ* + 1 clusters.

### Principal component analysis (PCA)

PCA was performed to visualize the differences between different autophagy-based molecular subclasses based on the expression pattern of autophagy-related genes. PCA plots were generated using FactoMineR package (v2.4).

### Survival analysis

Survival analysis was specifically focused on subgroups restricted to high and low expression groups based on the median expression of ATG4D, HIF1A, LAMP2, RPTOR, ULK1, and MAP1LC3B. All patients from GSE53786, GSE10846 and GSE181063 were divided into low-risk and high-risk groups based on the median risk score, and survival analyses were performed on these datasets. Survival was analyzed according to the Kaplan-Meier method, and differences between survival distributions were assessed with the log-rank test. Survival (v3.3-1) and Survminer (v0.4.9) packages were exploited to compute the survival curves and compare the differences between survival distributions.

### Differential expression and gene ontology analysis

The microarray data were analyzed using the limma package (v3.48.3) to screen for the DEGs between different autophagy-based molecular subclasses. Genes with adjusted P-values < 0.05 and log_2_|fold change| > 0.58 were considered as statistically significant differentially expressed genes. Gene Ontology including biological process, cellular component and molecular function analysis was carried using clusterProfiler package (v4.0.5) [23].

### Prognostic model construction

Differentially expressed genes (DEGs) between different autophagy-based molecular subclasses were initially subjected to a univariate Cox proportional hazard analysis to identify genes associated with survival. Genes with a P-value less than 0.002 from the analysis were subsequently subjected to a LASSO regression analysis for feature selection and model construction. The regression analysis was performed using the R-glmnet package (v4.1-3) [24], and the dataset was subsampled 1,000 times with replacement for this purpose. Identification of the optimal penalization coefficient lambda in the LASSO model was conducted through 10-fold cross-validation, adhering to the 1 standard error (1-SE) criterion [25].

The risk scores were calculated using the following formula:


$$\text{Riskscore}=\sum_{k=1}^{n}\left(\beta \text{k}\times \text{expression}(\text{gene}\;\text{k})\right)$$

Where n is the total number of genes in the model, βk is the coefficient of the kth gene, and expression (gene k) is the expression level of gene k.

### Model evaluation and validation

Following the construction of the prognostic model, its discriminative power was assessed in the training cohort (GSE53786) using time-dependent ROC curves at 1-year, 3-year, and 5-year time points, accompanied by the computation of area under the curve (AUC) values. Survival differences between patients with varying Riskscores were evaluated through survival analysis, as detailed in the “Survival Analysis” section. To ascertain the model’s generalizability and robustness, we extended our evaluation to two independent validation cohorts, GSE10846 and GSE181063, performing the aforementioned assessments.

### Screening for prognostic risk factors and constructing nomogram

A comprehensive nomogram was constructed by integrating the Riskscore model based on autophagy-related genes with other clinical prognostic factors for enhanced clinical application. Initially, potential prognostic factors, including the risk level derived from autophagy-based Riskscore model, gender, age, stage, ECOG scores, and molecular subtypes, were subjected to univariate Cox regression analysis. Following this, factors with a P-value of less than 0.05 in the univariate analysis were subsequently incorporated into the multivariate Cox regression for nomogram construction. Visualization of the nomogram was facilitated using the R-rms (v.6.2-0) and R-regplot (v1.1) packages.

### Analysis of immune infiltration and drug resistance and prediction of drug effectiveness for DLBCL patients

DLBCL patients were divided into high-risk and low-risk groups based on the established prognostic model. For immune infiltration analysis, R-cibersort package (v1.03) [26] was used to analyze the proportion of immune cells, employing the LM22 gene signature which can be accessed at https://cibersortx.stanford.edu/. Chemotherapeutic drug resistance of DLBCL patients was calculated using the pRRophetic package (v0.5) [27], with drugs retrieved from the cpg2016, encompassing a total of 237 drugs. The prediction of drug effectiveness was carried out using R-oncoPredict package (v0.2) [28], referencing two drug response datasets from the GDSC databases, including the GDSC1 dataset which contained 367 compounds and the GDSC2 dataset which contained 198 compounds, as of March 8, 2022. The differences in drug effectiveness between the two groups were compared by two-sided student’s *t*-test.

### Availability of data and materials

All the data and materials are available. Datasets used and/or analyzed in this study can be obtained from the corresponding author upon reasonable request.

## RESULTS

### Autophagy-related gene signatures stratify DLBCL into two molecular subtypes with prognostic differences

To explore the clinical significance of the autophagy-based Riskscore model and the prognostic characteristics of molecular subtypes in DLBCL based on autophagy-related gene signatures, the study was carried out according to the research flow chart (Supplementary Figure 1). We overlapped three databases: HADb, HAMdb, and AUTOPHAGY DATABASE, and acquired 80 autophagy-related genes (Supplementary Table 1). Heatmaps of gene expression profiles were across age, molecular subtypes, ECOG scores, and stages (Supplementary Figure 2). The differentially expressed autophagy-related genes were screened between patients < 60 years old and ≥ 60 years old, including TSC1 (P = 0.0484), BAG3 (P = 0.0472), and AMBRA1 (P = 0.035) (Supplementary Figure 2A). ABC, GCB and unclassified subtypes exhibited different expression of EGFR (*), EIF2S1 (*), WIPI2 (***), MTOR (*), ATG9A, BCL2, ITPR1, ULK2 (*), MAP1LC3B, GABARAPL1 (*), GABARAPL2 (*), ULK1 (*), CALCOCO2 (*), WIPI1, BAG3 (*), EIF2AK3 (**), FOXO3, GOPC (*), ATG2B (*), ERN1 (***), and AMBRA1 (Supplementary Figure 2B). Differential gene expression was also observed based on ECOG score classifications, with genes like CTSD (P = 0.0363), ULK2 (P = 0.0276), HDAC6 (P = 0.0425), PINK1 (P = 0.0225), SH3GLB1 (P = 0.0051), ULK1 (P = 0.0409), WDFY3 (P = 0.0327), BAG3 (P = 0.0427), SESN2 (P = 0.0409), MAP1LC3A (P = 0.0408), ERN1 (P = 0.0222), ATG9B (P = 0.0016) demonstrating variations (Supplementary Figure 2C). There was a statistical significance in ITPR1 (P = 0.0242) and EIF2AK3 (P = 0.0459) expression among patients with stage I, II, III, or IV DLBCL (Supplementary Figure 2D). These differences in autophagy-related genes across clinical factors prompted us to define novel molecular subtypes based on autophagy gene signatures.

**Figure 1:**
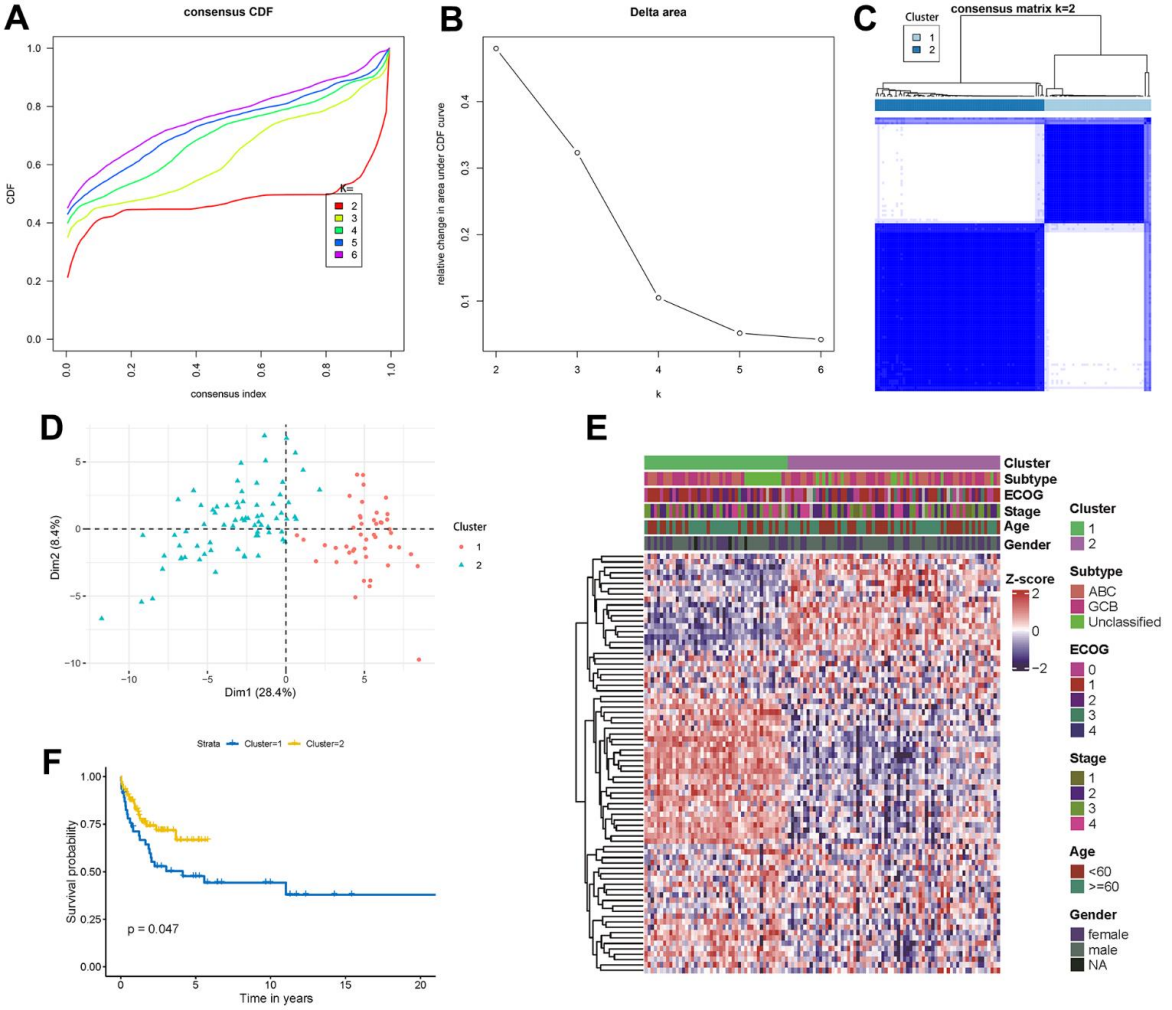
**Unsupervised gene expression analysis of the discovery set of 119 DLBCL.** (**A**) Consensus CDF with an increasing number of clusters (*k2* to *k6*); (**B**) Delta area plot displaying the relative changes in the area under the CDF curve; (**C**) Consensus matrix heatmap defining 2 clusters of samples for which consensus values range from 0 (white) to 1 (blue); (**D**) Principal component analysis of the 80 autophagy-related genes between cluster 1 and cluster 2; (**E**) Gene expression profiles heatmap of the 80 autophagy-related genes across the two molecular subtypes. ECOG, stage, age, and gender classifications were ordered in colored columns and rows corresponding to 80 autophagy-related genes; (**F**) Kaplan-Meier curves of overall survival in the 2 molecular subtypes. CDF, cumulative distribution function.

Differences in autophagy-related genes across clinical factors led us to explore novel molecular subtypes defined by autophagy gene signatures. Building on this, we conducted an unsupervised consensus analysis of 119 DLBCL samples, focusing on the expression profiles of the 80 autophagy-related genes. By achieving the clearest heatmap and highest cluster-consensus values (Supplementary Figure 3A, 3B), we classified patients into two clusters: cluster 1(n = 46, 38.66%) and cluster 2(n = 73, 61.34%) (Figure 1A, 1B). Clinicopathological characteristics among two molecular subtypes (cluster 1 and cluster 2) are summarized in Table 1, and were analyzed for statistical significance. Significant differences existed in age, ECOG scores, subtypes, and tumor TNM stages between the two cluster. PCA and consensus matrix confirmed the cluster distinction (Figure 1C, 1D). The expression patterns of autophagy-related genes were quietly different between cluster 1 and cluster 2 (Figure 1E). Differences in prognosis between the clusters were evident, with cluster 2 demonstrating a superior 5-year overall survival compared to cluster 1 (P = 0.047, Figure 1F).

### Autophagy genes associated with survival differences between molecular subtypes and their biological functions

We aimed to uncover the autophagy-related molecular mechanisms underlying the prognostic differences between the two molecular subtypes by identifying differentially expressed autophagy genes associated with survival and conducting a functional analysis. When comparing the transcriptome data from cluster 2 with cluster 1, we observed 6,276 up-regulated genes and 2,736 down-regulated genes in cluster 2. After excluding 768 non-coding genes, 8,244 protein-coding genes were retained for further examination (Supplementary Table 2). Annotation of gene ontology revealed that 8,244 DEGs were significantly implicated in the regulation of neuron projection development, protein maturation (Supplementary Figure 4A), cluster of actin-based cell projections, brush border (Supplementary Figure 4B), actin binding, active transmembrane transporter activity, active ion transmembrane transporter activity, and actin filament binding (Supplementary Figure 4C). Applying univariate Cox analysis on these 8,244 DEGs against patient survival, we identified 1,227 genes having a noteworthy association with survival (P < 0.05) (Supplementary Table 3). Among these, autophagy-related genes included ATG4D, RPTOR, ULK1, HIF1A, LAMP2, and MAP1LC3B. Patients stratified by the median expression of these 6 genes showed decreased ATG4D (P < 0.0035) (Supplementary Figure 4D), RPTOR (P < 0.0024) (Supplementary Figure 4G) and ULK1 (P < 0.0230) (Supplementary Figure 4H), and increased HIF1A (P < 0.0350) (Supplementary Figure 4E), LAMP2 (P < 0.0450) (Supplementary Figure 4F), and MAP1LC3B (P < 0.0670) (Supplementary Figure 4I) were associated with better 10-year overall survival. These results suggest that autophagy gene characteristics may be helpful in the prognostic assessment of DLBCL patients, prompting us to establish a prognostic model based on autophagy genes.

### Prognostic model establishment and evaluation

**Figure 2:**
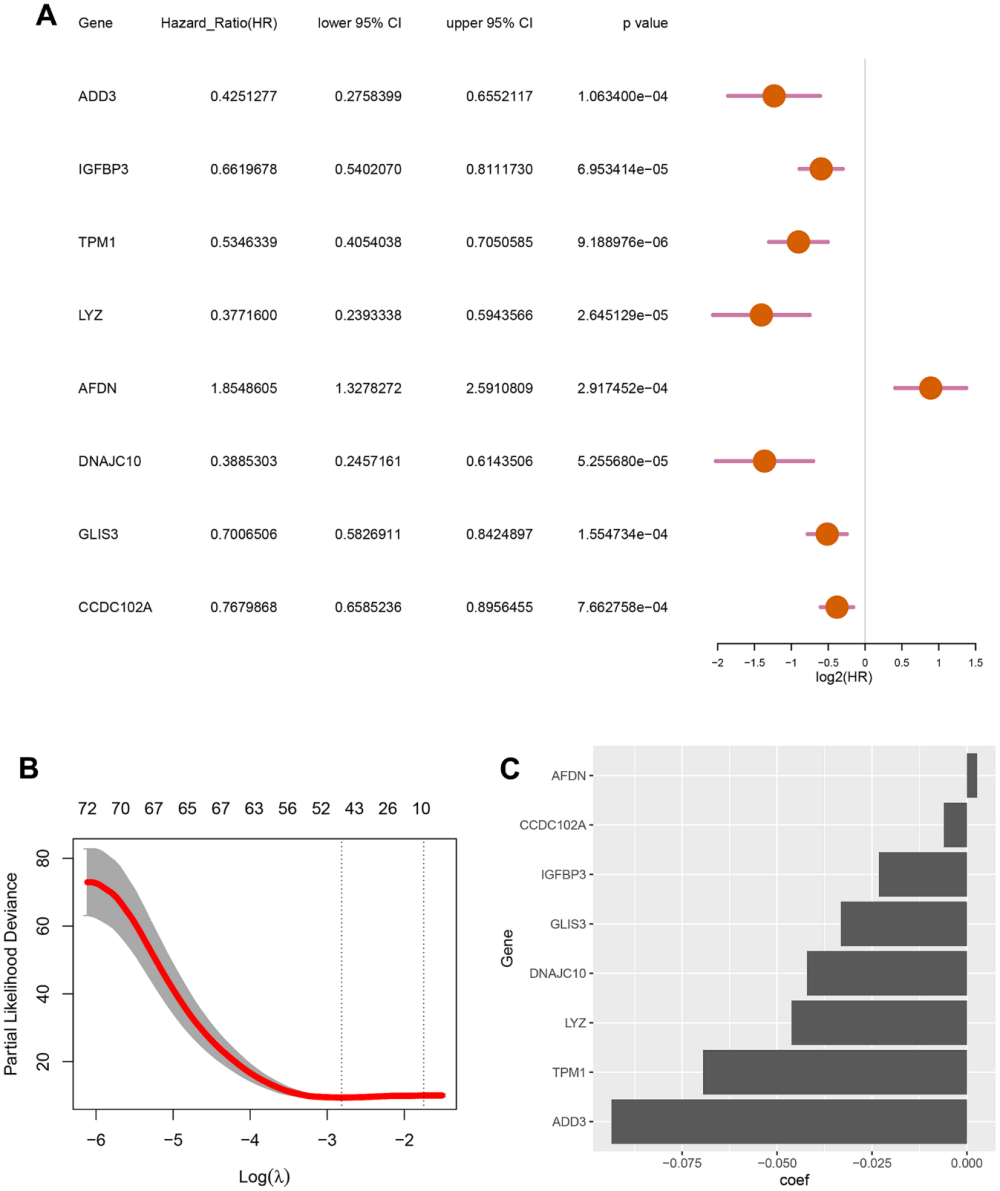
**Screening for autophagy-related genes associated with the survival probability of diffuse large B-cell lymphoma.** (**A**) Univariate Cox regression analysis for 8 autophagy-related genes correlated with the survival probability; (**B**) Partial likelihood deviance for the LASSO coefficients of 78 DEGs with the lambda as the tuning parameter; (**C**) Coefficient profiles of the 8 autophagy-related genes.

To develop a autophagy-based prognostic model for DLBCL patients, 78 genes with a P-value less than 0.002 from the previous univariate Cox regression analysis were subjected to LASSO regression. This identified eight autophagy-related genes to construct the prognostic model (Figure 2A). The optimum lambda value was confirmed as shown in Figure 2B. The coefficient distribution of the eight genes was presented in Figure 2C. Riskscore formula was as follows, Riskscore = 0.0027*E(AFDN)-0.0936*E(ADD3)-0.0232*E(TPM1)-0.0462*E(LYZ)-0.0421*E(DNAJC10)-0.0332*E(GLIS3)-0.0061*E(CCDC102A)-0.0232*E(IGFBP3). Where E indicates the expression level of the corresponding gene.

**Figure 3:**
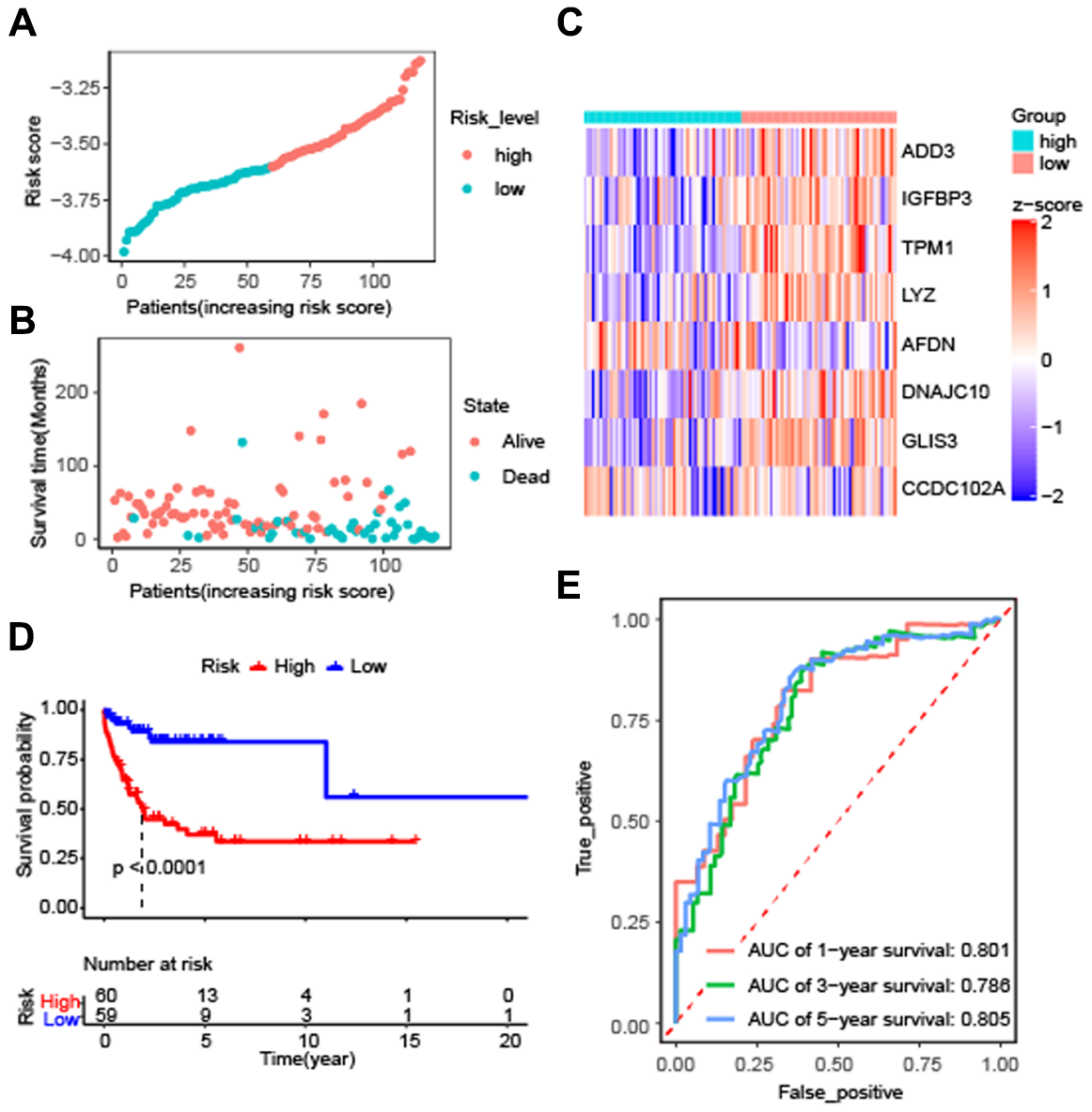
**Effectiveness evaluation of the prognostic model for patients with diffuse large B-cell lymphoma based on the data set GSE53786.** (**A**) The patients from GEO were divided into high-risk and low-risk groups based on the median of the risk scores; (**B**) The distribution of survival time of patients in alive or dead status; (**C**) The heatmap of 8 autophagy-related genes expression between high-risk and low-risk groups; (**D**) Kaplan-Meier curves of high-risk and low-risk groups (P < 0.0001 by the log-rank test); (**E**) The AUC scores at 1, 3, and 5 years.

To evaluate the effectiveness of the constructed model, the risk score and survival time distributions of the patients were analyzed. All patients were divided into high-risk and low-risk groups based on the median value of Riskscore, with those exceeding the median classified as high-risk and those below as low-risk (Figure 3A). The survival data-time plot is shown in Figure 3B, which indicates that deceased patients generally have higher risk scores. Moreover, patients with high risk were detected with low expression of ADD3, IGFBP3, TPM1, LYZ, AFDN, DNAJC10, GLIS3, and CCDC102A (Figure 3C). Kaplan-Meier survival curves presented that the patients with high risks exhibited significantly low survival probability (P < 0.0001) (Figure 3D). ROC curves were plotted and AUC scores of 1-year, 3-year, and 5-year survival were 0.801, 0.786, and 0.805, respectively (Figure 3E). These results implied that the risk prediction model based on autophagy-related genes can differentiate the prognosis of patients with DLBCL to a certain degree.

### External validation of the prognostic model

**Figure 4:**
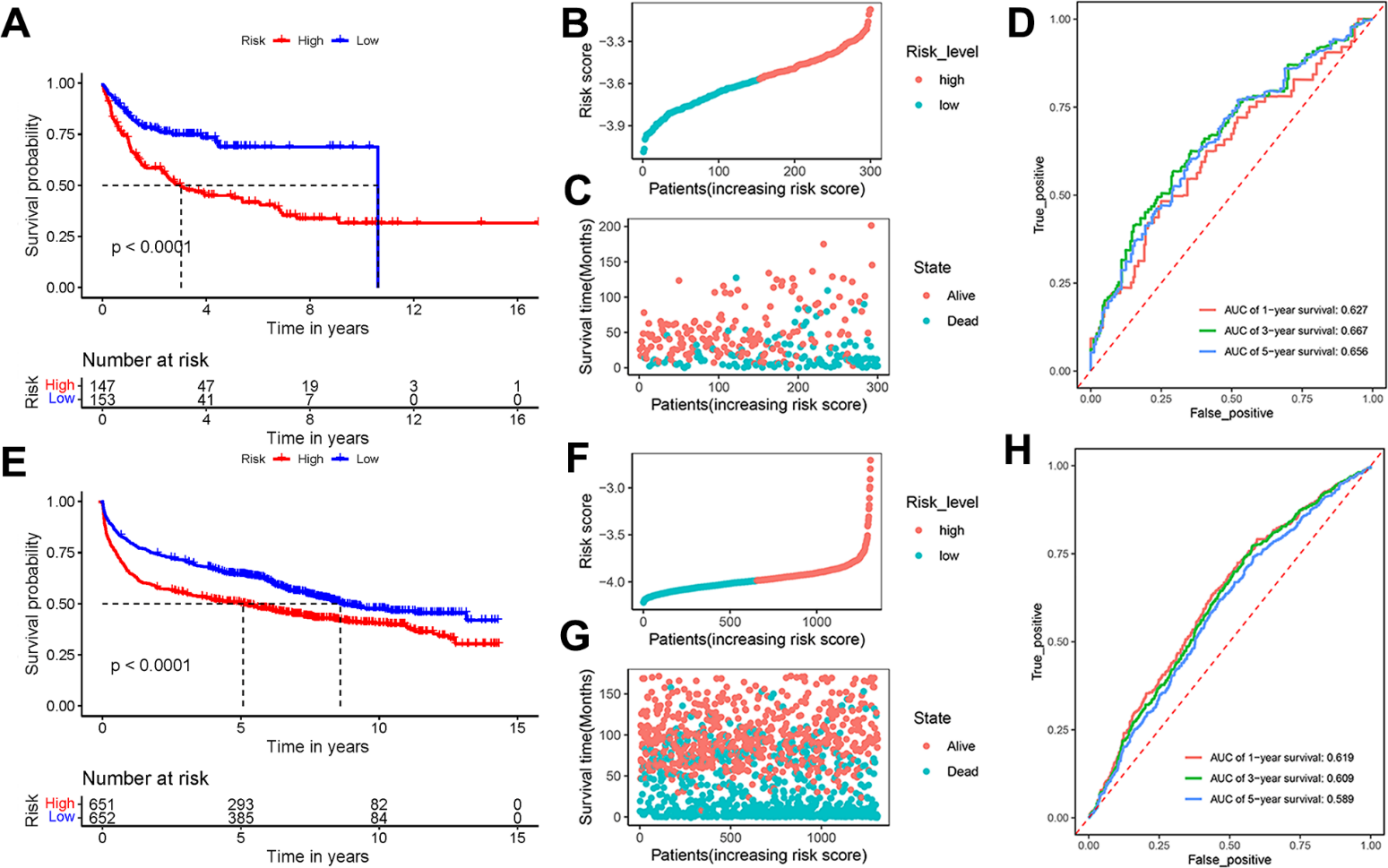
**Effectiveness evaluation of the prognostic model based on the external datasets GSE10846 and GSE181063.** (**A**) Kaplan-Meier curves, (**B**) risk score distribution, (**C**) survival time distribution, and (**D**) AUC scores at 1, 3, and 5 years in the dataset GSE10846; (**E**) Kaplan-Meier curves, (**F**) risk score distribution, (**G**) survival time distribution, and (**H**) AUC scores at 1, 3, and 5 years in the dataset GSE181063. Patients were divided into high-risk and low-risk groups based on the median of the risk scores.

To validate the universality and reliability of our model, we conducted evaluations using two separate validation datasets. The Kaplan-Meier survival analysis revealed that patients in the high-risk group had a lower survival probability than those in the low-risk group (P < 0.0001) (Figure 4A). Figure 4B presented the risk score distribution of 300 DLBCL patients in GSE10846 dataset. The survival time distribution showed that the patients in the alive status were predicted with low-risk scores and a long survival time (Figure 4C). AUC scores of 1-year, 3-year, and 5-year survival were 0.627, 0.667, and 0.656 in dataset GSE10846, respectively (Figure 4D). Analysis of GSE181063 dataset confirmed that patients with high-risk scores obviously had decreased survival probability (P < 0.0001) (Figure 4E). The risk score distribution of 1303 patients was shown in Figure 4F. Figure 4G confirmed the strong association between risk scores and survival time. For the GSE181063 dataset, AUC scores of 1-year, 3-year, and 5-year survival were 0.619, 0.609, and 0.589 respectively, as shown in Figure 4H. Considering the evidence from both datasets, we infer that our established model provides effective predictive insights for DLBCL patient outcomes.

### Prediction stability of the prognostic model for DLBCL patients with different clinical characteristics

Further assessing the stability of the prognostic model for DLBCL patients, we evaluated its performance in subgroups stratified by clinical factors. Patients were categorized into subgroups based on molecular subtypes (subtype ABC and GCB), gender (female and male), age (>= 60 and < 60), ECOG scores (>= 2 and < 2), and stage (I-II and III-IV). Survival probability was noticeably correlated with risk scores for both subtype ABC and GCB (Supplementary Figure 5A, 5B). Similarly, both female and male DLBCL patients with high-risk scores showed a lower survival probability than those with low-risk scores (Supplementary Figure 5C, 5D). For age, DLBCL patients >= 60 years and < 60 years in the low-risk group survived longer than those in the high-risk group (Supplementary Figure 5E, 5F). Consistent prognostic results were observed between DLBCL patients with ECOG >= 2 and ECOG < 2 (Supplementary Figure 5G, 5H), as well as between those staged I-II or staged III-IV (Supplementary Figure 5I, 5J). These results validated that the prognostic model’s accuracy for DLBCL patients was consistent across different clinical characteristics.

### Prognostic nomogram for overall survival of DLBCL patients

**Figure 5:**
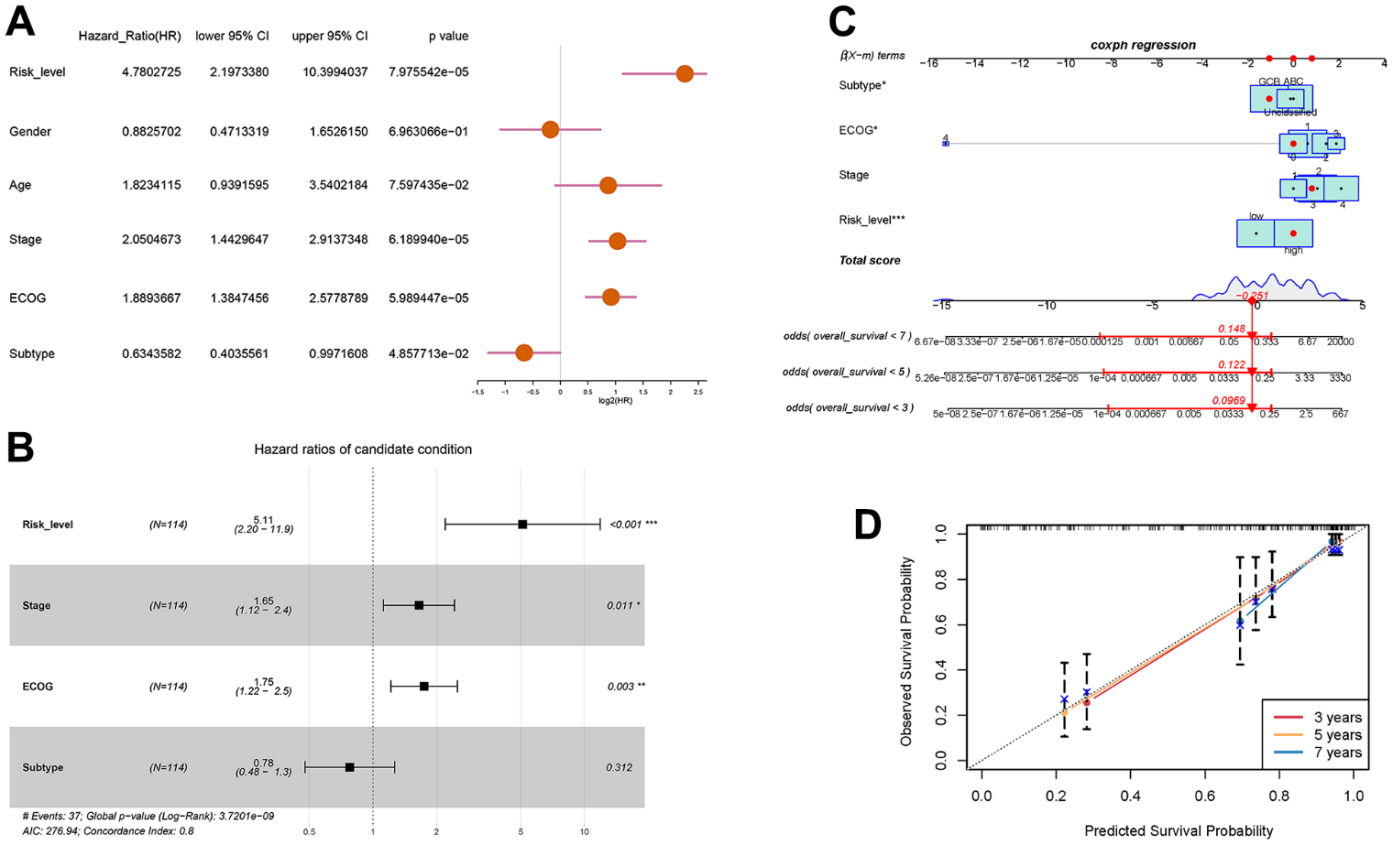
**Prognostic prediction of DLBCL patients based on clinical characteristics and risk scores of autophagy- and survival- related genes.** (**A**) Univariate Cox regression analysis of risk level, gender, age, stage, ECOG score, and subtype; (**B**) Multivariate Cox regression analysis of risk level, stage, ECOG, and subtype; (**C**) Nomogram for the prediction of 3-year, 5-year, and 7-year overall survival; (**D**) Calibration curves with nomogram-predicted 3-year, 5-year, and 7-year survival probability and observed survival frequency.

With the aim of enhancing the prediction and clinical utility for DLBCL prognosis, we constructed a nomogram incorporating both the autophagy-related prognostic model and clinical characteristics. Univariate analysis confirmed 4 significant prognostic factors, including risk level, stage, ECOG score, and molecular subtype (Figure 5A). Further multivariate Cox regression analysis screened the significant factors including risk level, stage, ECOG score, and molecular subtype (Figure 5B), which were then incorporated into the nomogram. Figure 5C showed the prognostic nomogram and odds values of overall survival at 3, 5, and 7 years. The calibration plot for 3-year, 5-year, and 7-year overall survival exhibited an optimal agreement between the observed outcomes and predictions by nomogram (Figure 5D). In summary, the nomogram seamlessly integrates multiple risk factors to offer individualized and precise prognostic predictions for DLBCL patients, enhancing its applicability in clinical settings.

### Immune infiltration and drug-resistance analysis for DLBCL patients with high-risk or low-risk

**Figure 6:**
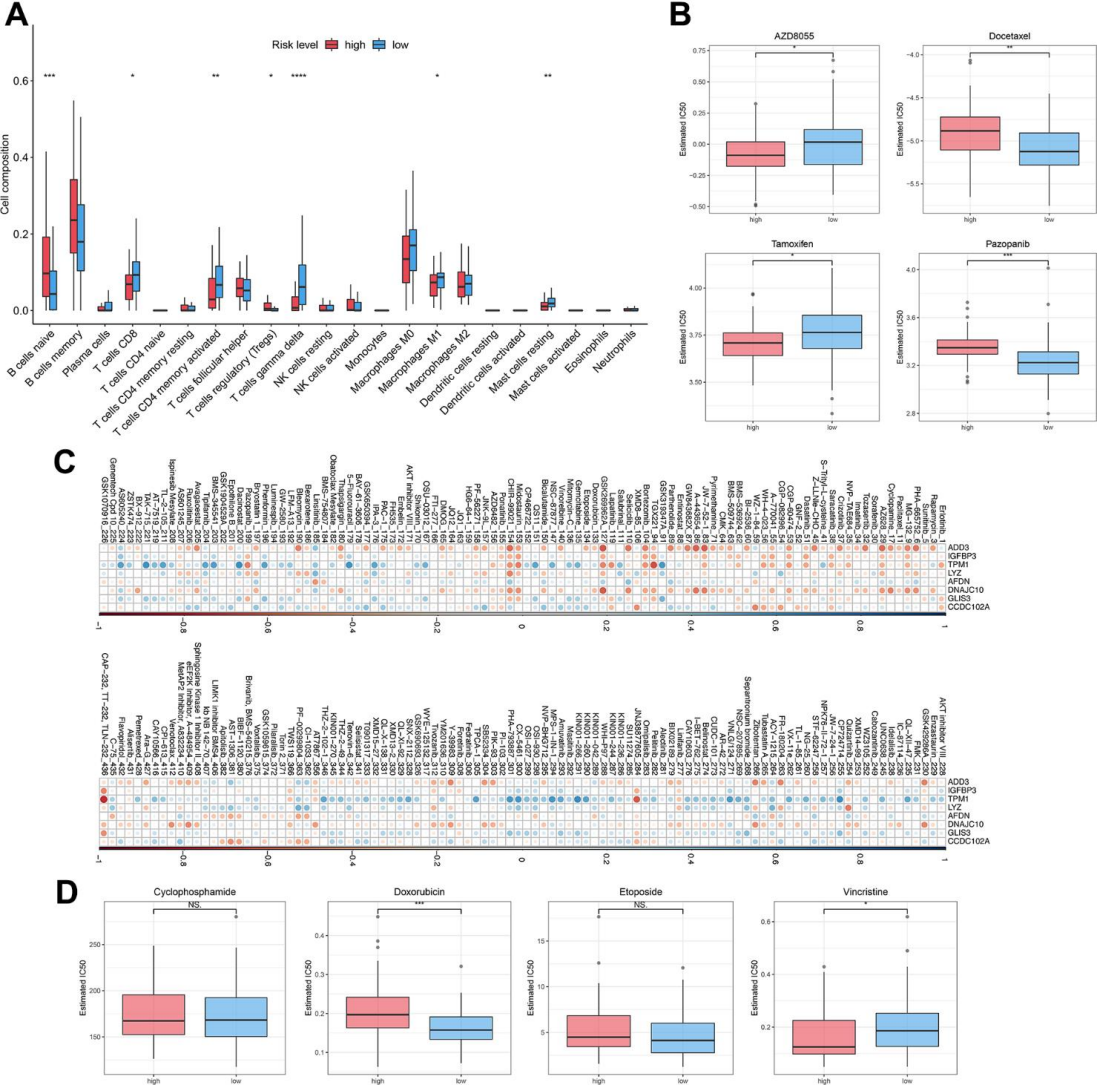
**Immune infiltration and drug sensitivity of DLBCL patients in the high-risk group or low-risk group.** (**A**) The immune infiltration of 22 leukocyte subtypes from DLBCL patients in high-risk and low-risk groups; (**B**) Individual IC50 values of AZD8055, tamoxifen, docetaxel, and pazopanib were shown; (**C**) Heatmap of the correlation coefficients for the risk factors and chemotherapeutic drugs; (**D**) Individual IC50 values of cyclophosphamide, doxorubicin, etoposide, and vincristine predicted for DLBCL patients in the high-risk and low-risk groups. nsP > 0.05, *P < 0.05, **P < 0.01, ***P < 0.001, ****P < 0.0001 (Two-sided student’s *t*-test).

To investigate the relationship between autophagy risk scoring, immune cell infiltration, and drug sensitivity, we performed immune cell infiltration analysis and drug sensitivity analysis. The abundance of immune cells were counted and depicted in Figure 6A. The proportion of native B cells (P < 0.001) and Treg (P < 0.05) cells was significantly increased in the high-risk group compared with the low-risk group. Conversely, DLBCL patients with high-risk scores exhibited lower proportions of CD8^+^ T cells (P < 0.05), CD4+ memory activated T cells (P < 0.01), gamma delta T cells (P < 0.0001), Macrophages M1 (P < 0.05), and resting mast cells (P < 0.01) than those in DLBCL patients with low-risk scores. In this study, we hypothesized drug resistance in DLBCL correlates with the risk status predicted by the prognostic model. Next, 119 DLBCL patients were divided into high-risk and low-risk groups and evaluated to ascertain potential resistance to 237 chemotherapeutic drugs. DLBCL patients in the low-risk group were estimated with higher IC50 of AZD8055 (P < 0.05) and tamoxifen (P < 0.05) and lower IC50 of docetaxel (P < 0.01) and pazopanib (P < 0.001) (Figure 6B) compared with patients in the high-risk group. Supplementary Table 4 lists the other chemotherapeutic drugs, of which drug resistance was associated with the risk status of DLBCL patients. Subsequent analyses focused on the relationship between drug resistance and risk factors such as ADD3, IGFBP3, TPM1, LYZ, AFDN, DNAJC10, GLIS3, and CCDC102A. It was found that the LYZ gene was positively correlated with drug resistance to most chemotherapeutic drugs. In contrast, the ADD3 gene was strongly negatively associated with drug resistance to JW-7-52-1, BEZ235, and A-443654 (Figure 6C). Lastly, the risk scoring model was then used for the prediction of drug efficiency using the drug information obtained from the GDSC database. Four chemotherapeutic agents commonly used for the treatment of DLBCL were analyzed here. It was shown that the estimated IC50 value of doxorubicin was higher in high-risk patients compared to low-risk patients (P < 0.001) (Figure 6D). In contrast, IC50 of vincristine in low-risk patients was obviously higher than in high-risk patients (P < 0.05). These results demonstrate the potential of the autophagy-related model in assessing the immune microenvironment of DLBCL and predicting drug sensitivity.

## DISCUSSION

Patients with DLBCL have different survival rates considering the heterogeneity of the diseases [29]. Current efforts have advanced in understanding the genome and transcriptome of DLBCL that distinguish subgroups of patients with poor prognosis after chemo-immunotherapy [30–33]. Autophagy-related gene expression profiles are believed to delineate two distinct groups of cases with high or low risks in terms of survival probability [34]. However, further construction and validation of the prediction method are required before the clinical transformation. Hence, this study established a prognostic assessment model based on the expression profile of autophagy-related genes and clinical characteristics of DLBCL patients. The established risk score method was then applied to predict immune infiltration and drug resistance, which may provide clinical potential in therapeutic interventions for individual cases.

Autophagy manifests as an adaptive response to stress stimuli and an intracellular degradative pathway in cancer upon oxygen deficiency, nutrition shortage, and even chemotherapies. Experimental studies have confirmed that autophagy regulators like BCL-2 and BECN1 mediate autophagy responses contributing to lymphomagenesis [35–37]. Transcriptomic studies excavated the expression signatures of 25 autophagy- and survival-related genes [38].

Within distinct autophagic molecular subtypes, our research pinpointed six differentially expressed autophagy genes that are closely associated with survival: ATG4D, HIF1A, LAMP2, RPTOR, ULK1, and MAP1LC3B. These genes have been recognized to mediate the activation of autophagy in carcinoma tumorigenesis [39–44]. Among them, ATG4D, a member of the autophagy-related protein 4 (ATG4) family, serves as an intriguing nexus between autophagy and apoptosis across multiple cancers. Notably, anomalies in ATG4D promoter methylation and its defective expression have been correlated with a suppression of the autophagy signaling pathway, as evidenced in invasive ductal carcinoma and human uterine fibroids [38, 39]. In another study on hepatocellular carcinoma, elevated ATG4D expression in tumor tissues was found, and its silencing led to decreased cell proliferation and increased sensitivity to cisplatin [45]. HIF1A, responsible for cellular responses to hypoxia, triggers autophagy in solid tumors under low oxygen conditions. This hypoxia-induced autophagy is known to diminish radiosensitivity and resistance to photodynamic therapy via the HIF1A-related pathway in colon cancer cells [46, 47]. Moreover, HIF-1α augments autophagy by modulating the expression of BNIP3, a protein central to stress adaptation mechanisms that fortify tumor cell survival while sidestepping apoptosis [48]. In addition, HIF-1α has been found to exert a tumor-promoting role in prostate cancer via affecting autophagy [49]. LAMP2, a ubiquitously expressed glycosylated protein found primarily on lysosome membranes, is essential for the correct fusion of autophagosomes with lysosomes [50]. Within the cancer landscape, studies have shown that LAMP2 plays a role in cell survival and disease progression. For instance, in neuroendocrine prostate cancer, knockdown of LAMP2 by siRNA induced an autophagy blockade and decreased both cancer cell proliferation and neuroendocrine markers [51]. An increased expression of LAMP2 in salivary adenoid cystic carcinoma, which was associated with cancer progression [52]. In addition, a reduced expression of LAMP2 has been associated with a decreased resistance to both cisplatin in human ovarian carcinoma cells and azacitidine in acute myeloid leukemia [53]. RPTOR (Raptor) is a protein that is part of the mTORC1 complex that negatively regulates autophagy. RPTOR/ULK1/autophagy axis influence esophageal squamous cell carcinoma tumorigenesis [54]. Unc-51-like kinase 1 (ULK1) is a serine/threonine kinase that participates in the initiation of autophagy. It plays a critical role in initiating autophagy and has been implicated in cancer drug resistance, as reviewed in recent literature [55]. Research has highlighted its role in cancer cell survival and pinpointed it as a potential therapeutic target [56]. Numerous studies have emphasized that altering ULK1 can impact both autophagy and apoptosis, affecting the trajectory of cancer growth and its sensitivity to treatments [57, 58]. Emerging evidence suggests that ULK1’s regulation could be crucial for overcoming drug resistance in various cancer types [59, 60]. MAP1LC3B (Microtubule-associated protein 1 light chain 3 beta) is a protein that plays a central role in the autophagy pathway, where it functions in autophagy substrate selection and autophagosome biogenesis. Studies have shown that MAP1LC3B and its adaptor sequestosome 1 (SQSTM1) modulate autophagy for tumorigenesis and prognosis in certain subsites of oral squamous cell carcinoma [61]. Elevated MAP1LC3B expression aligns with worse outcomes in gastric cancer patients [62]. In summary, these genes play vital roles in regulating autophagy activation, autophagy-apoptosis interplay, lysosome function, mTORC1 signaling, autophagy initiation, and autophagosome formation in the context of cancers. Modulating these genes could impact cancer cell proliferation, drug resistance, treatment response, and prognosis. Further investigation into their functional mechanisms may shed light on more precise immunotherapy approaches for DLBCL.

We aimed to elucidate the molecular mechanisms that account for the prognostic disparities between the two autophagy-related molecular subtypes by conducting GO enrichment analysis, in which certain biological processes were found to be enriched. Notably, actin-related structures and functions, such as clusters of actin-based cell projections, actin binding, and actin filament binding, were prominently enriched. Actin is pivotal in maintaining cell shape and participates in vital cellular functions, including movement and division. Alterations in actin dynamics are linked to cancer metastasis. Specifically, DLBCL has been associated with elevated levels of phosphorylated actin-binding proteins, namely Ezrin-Radixin-Moesin (ERM) [63]. The synergy between BCR signaling and the actin cytoskeleton underpins the innate regulation of B cells [64, 65]. In the realm of hematological malignancies, mutations in actin genes ACTB and ACTG1 are predominantly linked to lymphoid cancers [66]. RhoA, a Rho GTPase and regulator of actin-based cytoskeletal dynamics, is recognized as driver gene in DLBCL [67]. This evidence suggests that actin-related structures and functions could be instrumental in the onset and progression of DLBCL. Additionally, cellular pathways associated with transmembrane transporter activity, encompassing active transmembrane transporter activity and active ion transmembrane transporter activity, were also enriched. Glucose transporter 3 (GLUT3) is posited as a potential prognostic marker in DLBCL. Elevated expression levels of GLUT-3 in DLBCL patients correlate with diminished progression-free survival (PFS) [68]. In the cancer milieu, transmembrane diffusion can modulate the intake and expulsion of anticancer drugs, thereby impacting their potency and resistance. ATP binding cassette (ABC) transporters are a superfamily of transmembrane proteins involved in the active transport of a wide range of substrates, including drugs, across cell membranes. These pathways are involved in the transport of molecules across the cell membrane, which is essential for maintaining cellular homeostasis and proper functioning of cells. The dysregulation of transmembrane transporter activity can disrupt cellular processes and contribute to the development and progression of diseases, including cancer. Dysregulation of ABC transporters has been associated with multidrug resistance in cancer cells, limiting the effectiveness of chemotherapy. Interestingly, variations in the excretion via transmembrane transporters ABCB1 might forecast the therapeutic efficacy of lenalidomide in Mantle Cell Lymphoma [69]. Thus, the enrichment of transmembrane transporter activity pathways in DLBCL could be a factor in the observed drug resistance, aggressive tendencies, and adverse outcomes of this malignancy. In summary, these insights offer fresh avenues to delve into the autophagy-related molecular and cellular dynamics pertinent to DLBCL’s pathogenesis and behavior. In our recent efforts to understand the complex landscape of DLBCL and its varied prognosis, we developed a prognostic model centered on the role of autophagy, a process that has been increasingly recognized for its significance in cancer biology.

The 8 genes selected in our study included ADD3, IGFBP3, TPM1, LYZ, AFDN, DNAJC10, GLIS3, and CCDC102A, which are predominant aspects of autophagy regulation in pathophysiological status of a large number of cancers [70–76]. Specifically, genes like ADD3 and LYZ have been previously implicated in potential roles related to the progression and metastasis of certain cancers [77, 78]. The expression of IGFBP3 and TPM1, on the other hand, has been closely associated with tumor cell growth and survival [79, 80], further emphasizing the potential significance of these genes in the context of DLBCL.

While there are several prognostic models available for DLBCL, the uniqueness of our model stems from its concentrated focus on autophagy-a pivotal yet relatively uncharted domain in DLBCL’s pathogenesis. Autophagy plays a central role in cellular survival, proliferation, and death. A deeper understanding of this process is paramount for grasping the biology of DLBCL and formulating effective therapeutic strategies. Another notable feature of our model is its robust AUC values across diverse datasets, which not only validate the model’s superiority but also underscore its potential in predicting the prognosis of DLBCL patients. This not only highlights the potential value of our model in prognostic predictions for DLBCL patients but also suggests its broader applicability in future clinical practices. In conclusion, our model, rooted in an in-depth study of autophagy-related genes, offers a novel and more precise prognostic tool for DLBCL. It paves the way for valuable insights into future therapeutic strategies, emphasizing the importance of autophagy in the disease’s biology.

DLBCL is a heterogeneous lymphoma characterized by the infiltration of various immune cells within the tumor microenvironment. The composition and presence of these immune cell infiltrates have been linked to prognostic implications in DLBCL [81]. Our risk prediction model, predicated on autophagy-related genes, offers novel insights into the immune landscape of DLBCL patients. Notably, DLBCL patients with high-risk scores exhibited lower proportions of CD8^+^ T cells, CD4^+^ memory activated T cells, gamma delta T cells, Macrophages M1, and resting mast cells, while showing elevated levels of native B cells and regulatory T (Treg) cells. Native B cells, which are the malignant cells in DLBCL, play a central role in the pathogenesis of the disease. DLBCL is characterized by the clonal expansion of B cells that have undergone genetic alterations, leading to uncontrolled proliferation and survival. Treg cells, a subset of CD4+ T cells with immunosuppressive functions [82], have been found to be associated with poor prognosis in DLBCL [83]. The increased presence of Treg cells in high-risk DLBCL patients suggests their potential role in suppressing anti-tumor immunity, further exacerbating the disease’s progression. Compared to healthy individuals, DLBCL patients, especially those at high risk, exhibited decreased counts of CD3+, CD4+, and CD8+ T cells, as well as natural killer (NK) cells [84]. Remarkably, the proportion of CD4+ and CD8+ T cells increased in these patients after treatment [85]. Moreover, a recent study demonstrated that higher CD8+ T cell levels were associated with improved immunotherapy outcomes in DLBCL [86]. Recent studies have emphasized the central role of CD4 T cells in peripheral tolerance, immunosuppression, and anti-tumor immunity. Their activation in DLBCL is indicative of a better prognosis [87]. However, their reduced infiltration in high-risk patients could weaken the overall immune response. Gamma delta T cells, a subset of T cells expressing the gamma delta T cell receptor, have been found to be enriched in DLBCL. While their exact role remains elusive, their presence hints at a potential role in the immune response against the tumor. In DLBCL, γδ T cells make up a significant portion of infiltrating T lymphocytes, with the non-GCB subtype showing a reduced frequency of these cells [88]. Given that γδ T cells can be activated by B-cell lymphoma, they are crucial in anti-tumor responses against B-cell malignancies and are promising targets for immunotherapies in DLBCL [89]. Their diminished presence in high-risk patients suggests a potential compromised immune defense against the tumor, emphasizing the need for therapeutic strategies targeting these cells. M1 macrophages, a subtype of macrophages with anti-tumor properties, have been associated with better prognosis in DLBCL. They can bolster the activation of cytotoxic T cells and amplify the immune response against cancer cells. Gene expression profiles from DLBCL biopsy specimens have shown an increased infiltration of macrophages [90]. Marinaccio et al. [91] demonstrated opposing roles of inhibition and promotion of angiogenesis based on the M1 and M2 phenotypes of TAM, M1 macrophage having antitumor and antiangiogenic roles. However, their reduced presence in high-risk patients might hinder the overall anti-tumor immune response, emphasizing their role in disease progression and treatment. Mast cells are known to play a role in inflammation and immune response, and they can be activated by various signals in the tumor microenvironment. A study on hepatocellular carcinoma found high resting mast cell infiltration patients have better outcome [92]. However, the role of mast cells in DLBCL is not well characterized, but their presence suggests a potential involvement in the tumor microenvironment. Recent findings have shown a marked increase in tryptase-positive mast cells, typically deemed activated, in chemo-resistant non-responder DLBCL patients compared to chemo-sensitive responders [93]. In summary, the increased regulatory T (Treg) cells along with decreased CD8+ T cells, CD4+ memory activated T cells, gamma delta T cells, M1 macrophages, and resting mast cells in high-risk DLBCL patients may facilitate tumor progression by hampering overall anti-tumor immune responses, increasing chemoresistance, etc., leading to poorer prognosis in this subset of patients. The altered immune landscape in high-risk patients emphasizes the need to understand the interactions between immune cells and the DLBCL tumor microenvironment. Our study provides novel insights into the role of autophagy in modulating this microenvironment. Utilizing the autophagy risk score could refine therapeutic decisions, enhancing treatment precision and efficacy. Differences in immune cell proportions can illuminate varied immune responses among DLBCL patients across risk spectrums, holding significant prognostic value and influencing therapeutic approaches. Envisioning personalized immunochemotherapy regimens becomes plausible by considering each patient’s unique immune profile. Furthermore, the balance between immune recognition and tumor evasion may be influenced by the autophagy-related genes we identified. Through our autophagy risk score model, we can predict this balance more accurately, underscoring the significance of our model in risk stratification and therapeutic outcomes.

Autophagy, a cellular self-degradation mechanism, plays a pivotal role in modulating the response of DLBCL cells to anticancer treatments. Notably, a study on Myc-induced lymphoma demonstrated that inhibiting autophagy, either through chloroquine or ATG5 shRNA, augmented the impact of p53 activation and alkylating drug therapies, leading to enhanced tumor cell apoptosis. This finding underscores the potential of autophagy suppression as a therapeutic strategy to potentiate apoptosis-inducing treatments in DLBCL. Further research has illuminated that autophagy inhibition can bolster the effectiveness of alkylating drugs, especially in tumors that are resistant to apoptosis. Several molecules such as bortezomib and antimalarial artemisinin derivative SM1044 indicate the beneficial potential to inhibit the progress of DLBCL through mediating autophagy [17]. This suggests a promising avenue to address drug resistance challenges in DLBCL. We confirmed that the selected genes LYZ was positively associated with the drug resistance to most chemotherapeutic drugs, while ADD3 was negatively related to the drug resistance to JW-7-52-1, BEZ235, and A-443654. Besides, the predicted IC50 values showed differences between low-risk and high-risk patients. These findings further validate the association between autophagy and drug sensitivity in DLBCL. Moreover, our autophagy-related model demonstrates potential in predicting drug sensitivity, offering a direction for targeted autophagy-based antitumor therapies.

Our study, while offering groundbreaking insights into the role of autophagy in DLBCL and introducing a novel predictive model, does come with certain limitations. The sample size, though substantial, may not fully capture the intricate heterogeneity of DLBCL, potentially limiting the generalizability of our findings. Additionally, our conclusions, which are primarily grounded in computational analyses, lack functional validation. This makes it imperative that the model-based immunotherapy also needs to be verified by *in vitro* and *in vivo* experiments in the future. As we look ahead, it’s paramount to validate the predictive accuracy of our model in larger and more diverse patient cohorts. A deeper exploration into the molecular mechanisms that underpin our model’s predictions will not only solidify our understanding but also spotlight potential therapeutic targets. Furthermore, refining our model by incorporating a broader spectrum of biomarkers or clinical parameters could enhance its predictive power and clinical utility. In essence, while our findings mark a significant step forward, the journey towards translating these insights into clinical practice requires continued rigorous research and validation.

## CONCLUSIONS

Summarily, the expression signature of autophagy-related genes was statistically different among DLBCL groups with different clinical features including age, molecular subtypes, ECOG scores, and stages. Consensus unsupervised analysis revealed 2 clusters of DLBCL samples based on 80 autophagy-related genes. The risk scoring model was constructed based on LASSO regression analysis, of which accuracy was externally evaluated. Prognostic nomogram for overall survival of DLBCL patients incorporated risk level, stage, ECOG scores, and molecular subtypes, exhibiting an optimal agreement between the actual observation and predicted results. The risk scoring method applies to the analysis of immune infiltration and drug resistance for DLBCL patients.

## Supplementary Material

Supplementary Figures

Supplementary Table 1

Supplementary Table 2

Supplementary Table 3

Supplementary Table 4
