# Supplementary material for: Prediction significance of autophagy-related genes in survival probability and drug resistance in diffuse large B-cell lymphoma
Source: Aging (Albany NY). 2024 Jan 17;16(2):1049–76. doi: 10.18632/aging.205282 (PMC10866451; doi:10.18632/aging.205282)
Supplement: Supplementary Figures [file aging-16-205282-s001.pdf]

SUPPLEMENTARY FIGURES

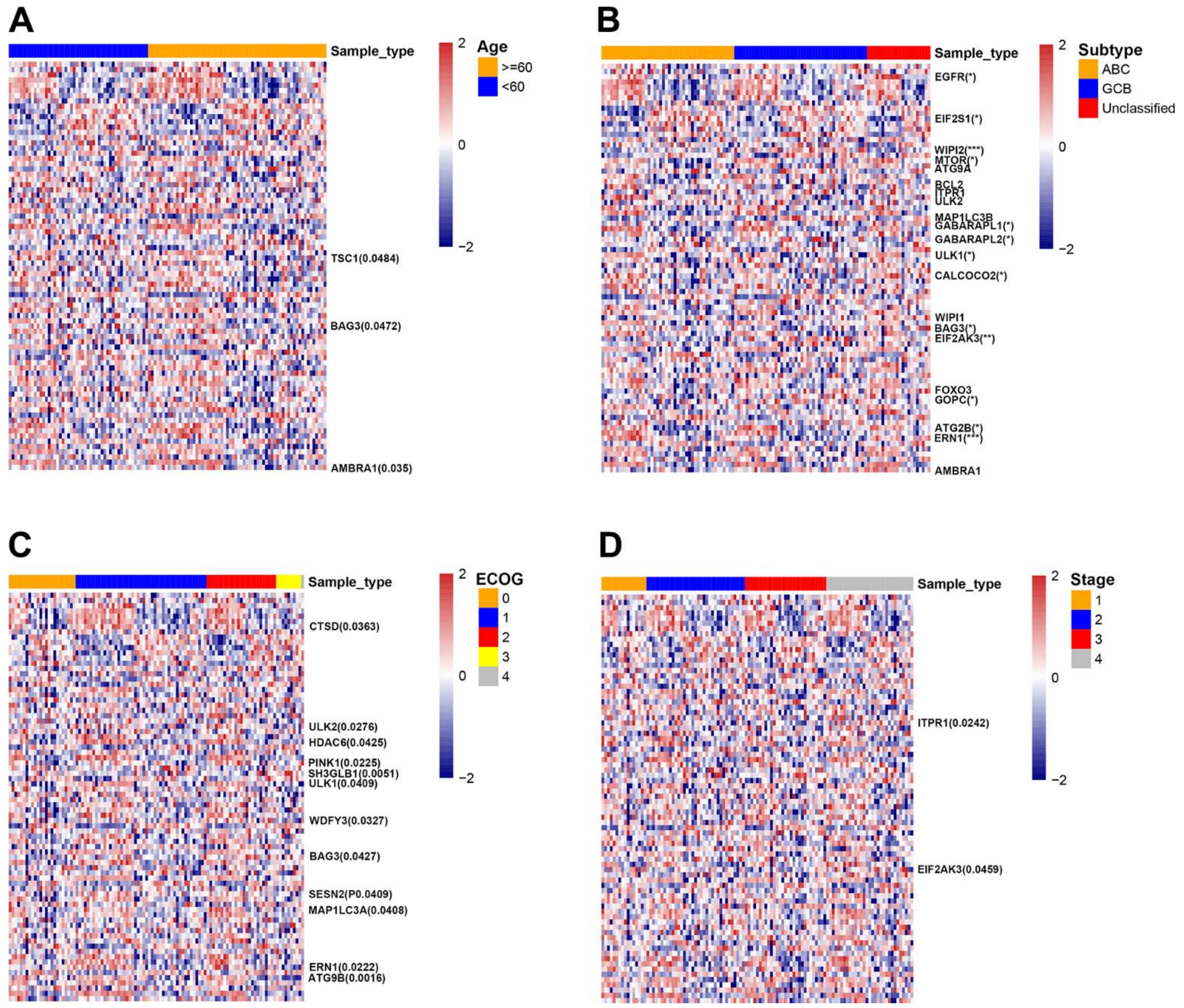

Supplementary Figure 1. Heatmap of alterations in genetic expression profiles across. (**A**) age, (**B**) subtype, (**C**) ECOG, and (**D**) stage. Columns correspond to tumor samples and rows correspond to 80 autophagy-related genes.

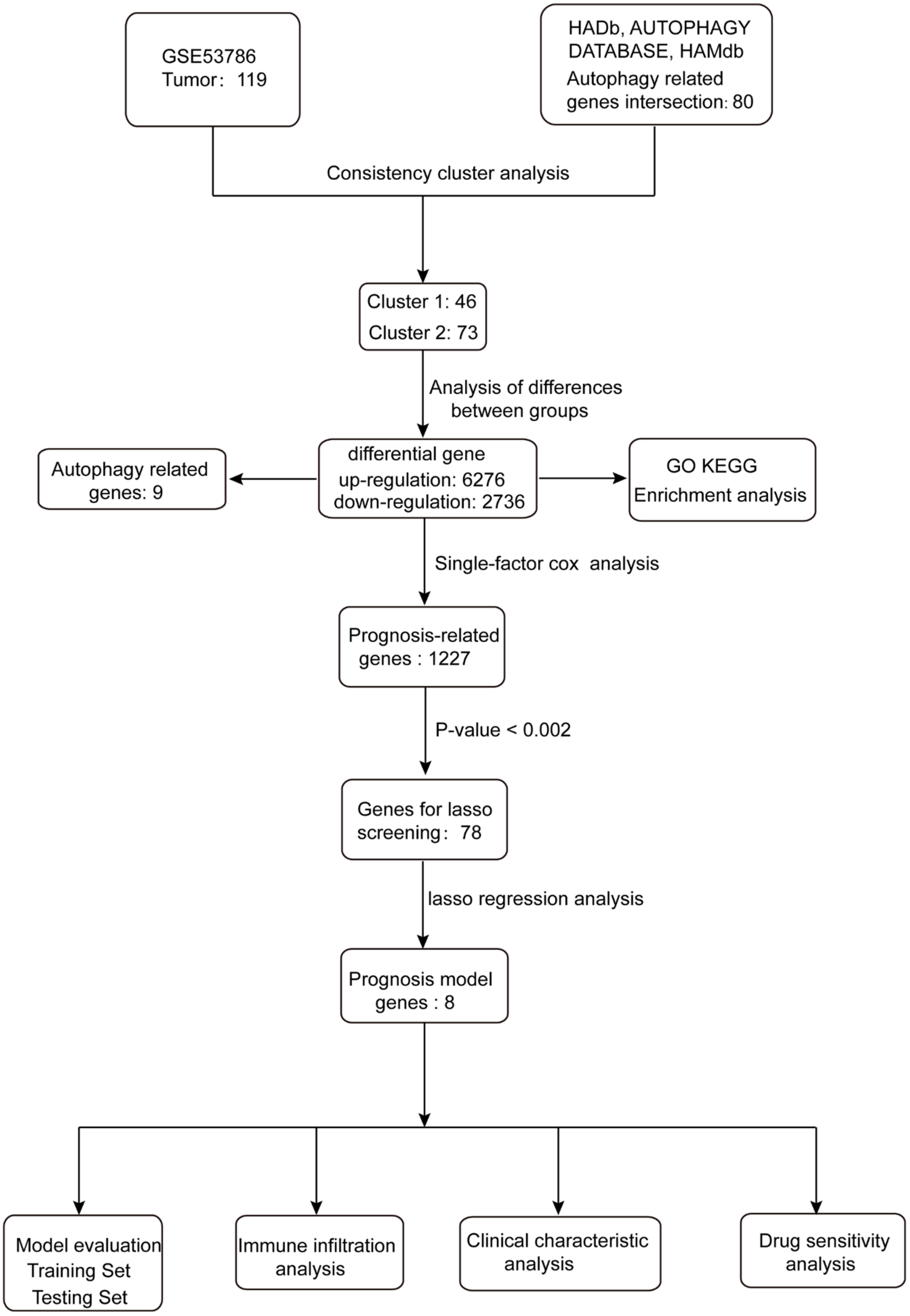

Supplementary Figure 2. The workflow of this study.

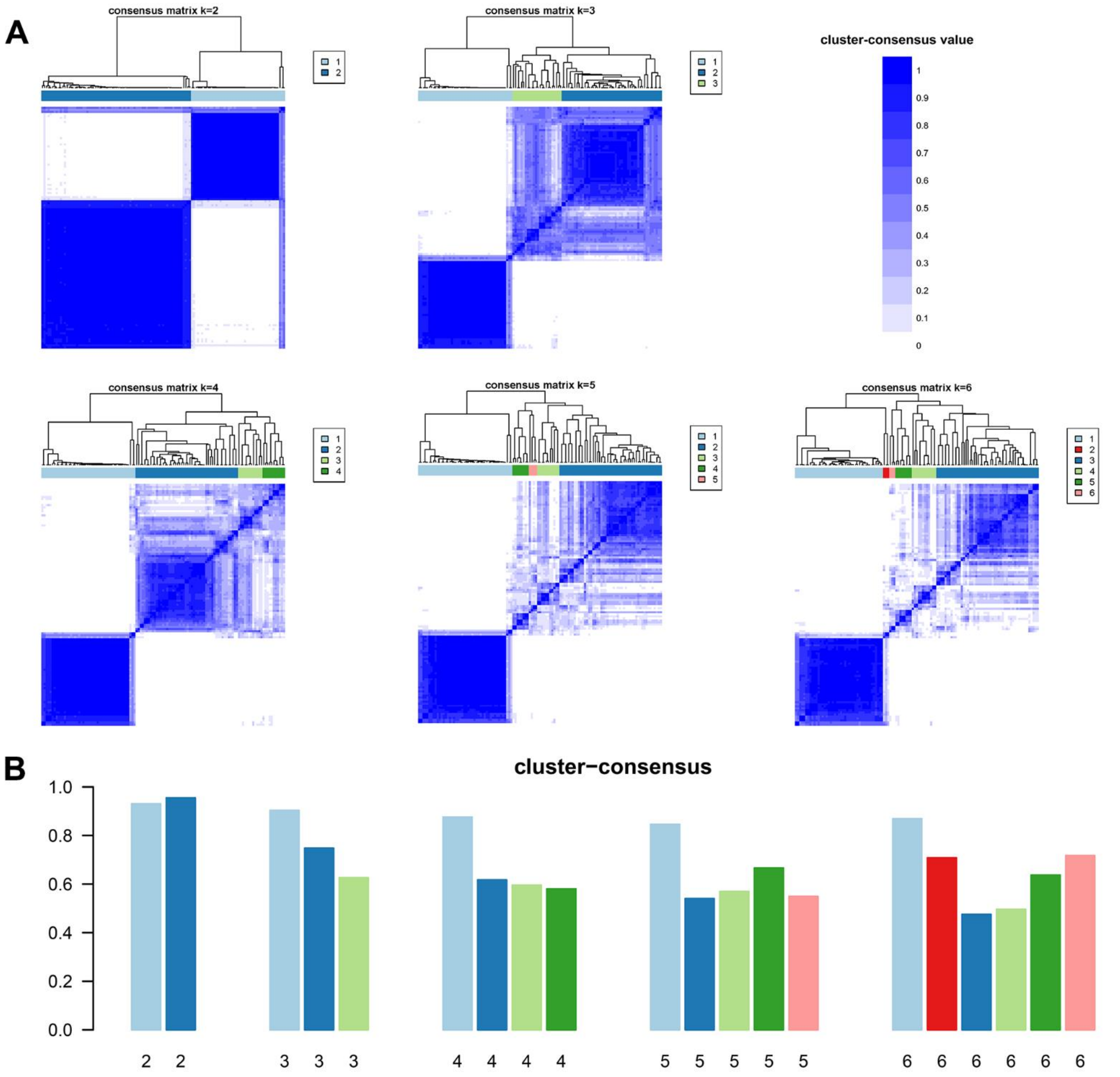

Supplementary Figure 3. Unsupervised clustering molecular subtypes of DLBCL based on autophagy genes. (**A**) Consensus matrix heatmap depicting consensus values for different numbers of clusters. (**B**) The cluster-consensus values for different numbers of clusters.

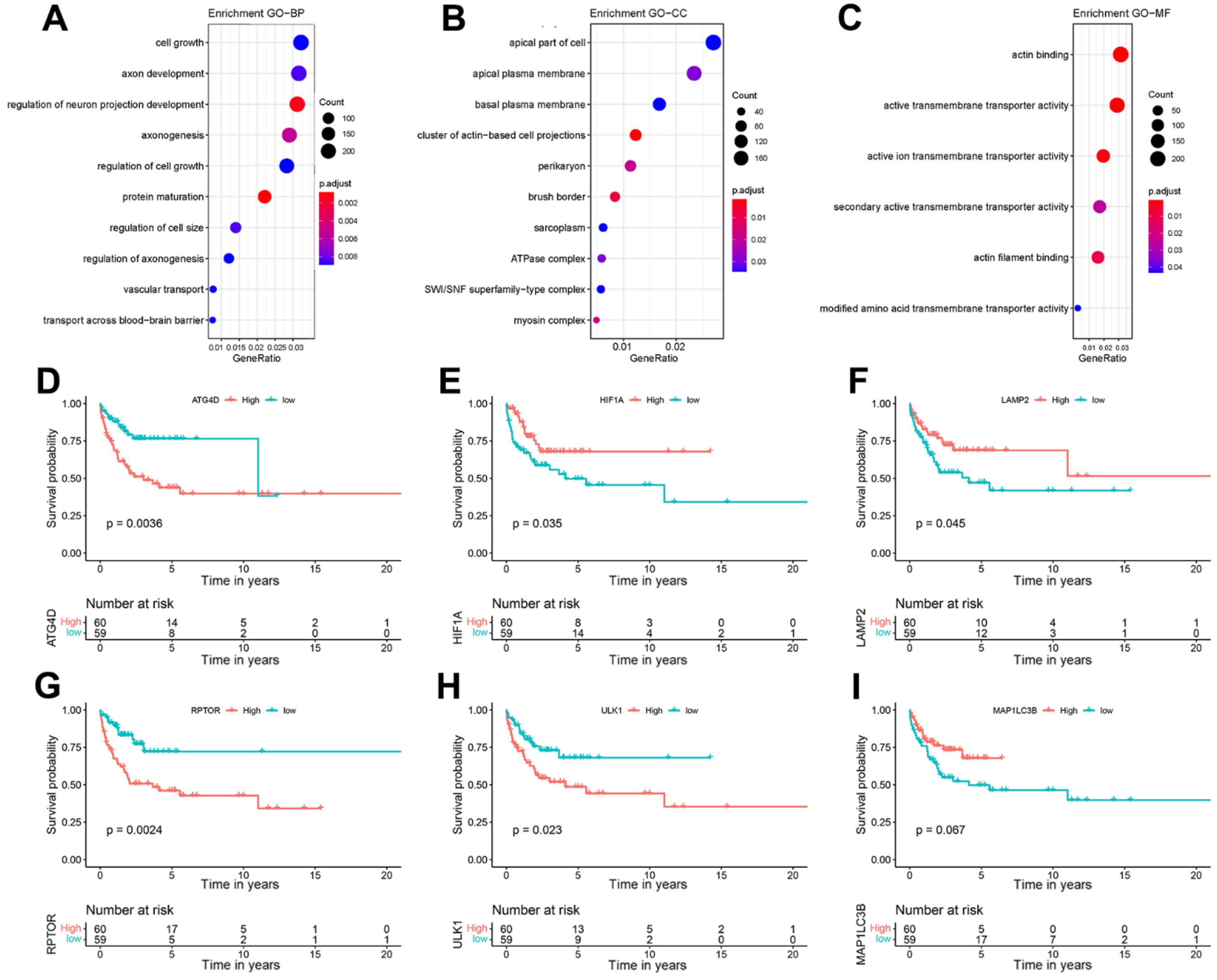

Supplementary Figure 4. GO annotation of the differentially expressed genes and survival analysis of DLBCL patients. GO enrichment analysis includes (**A**) biological process, (**B**) cell component, (**C**) molecular function. Prognosis signatures of DLBCL patients with high or low expression of (**D**) ATG4D, (**E**) HIF1A, (**F**) LAMP2, (**G**) RPTOR, (**H**) ULK1, and (**I**) MAP1LC3B; the median of gene expression was used as a cut-off for survival analysis. GO, gene ontology; DLBCL, diffuse large B-cell lymphoma.

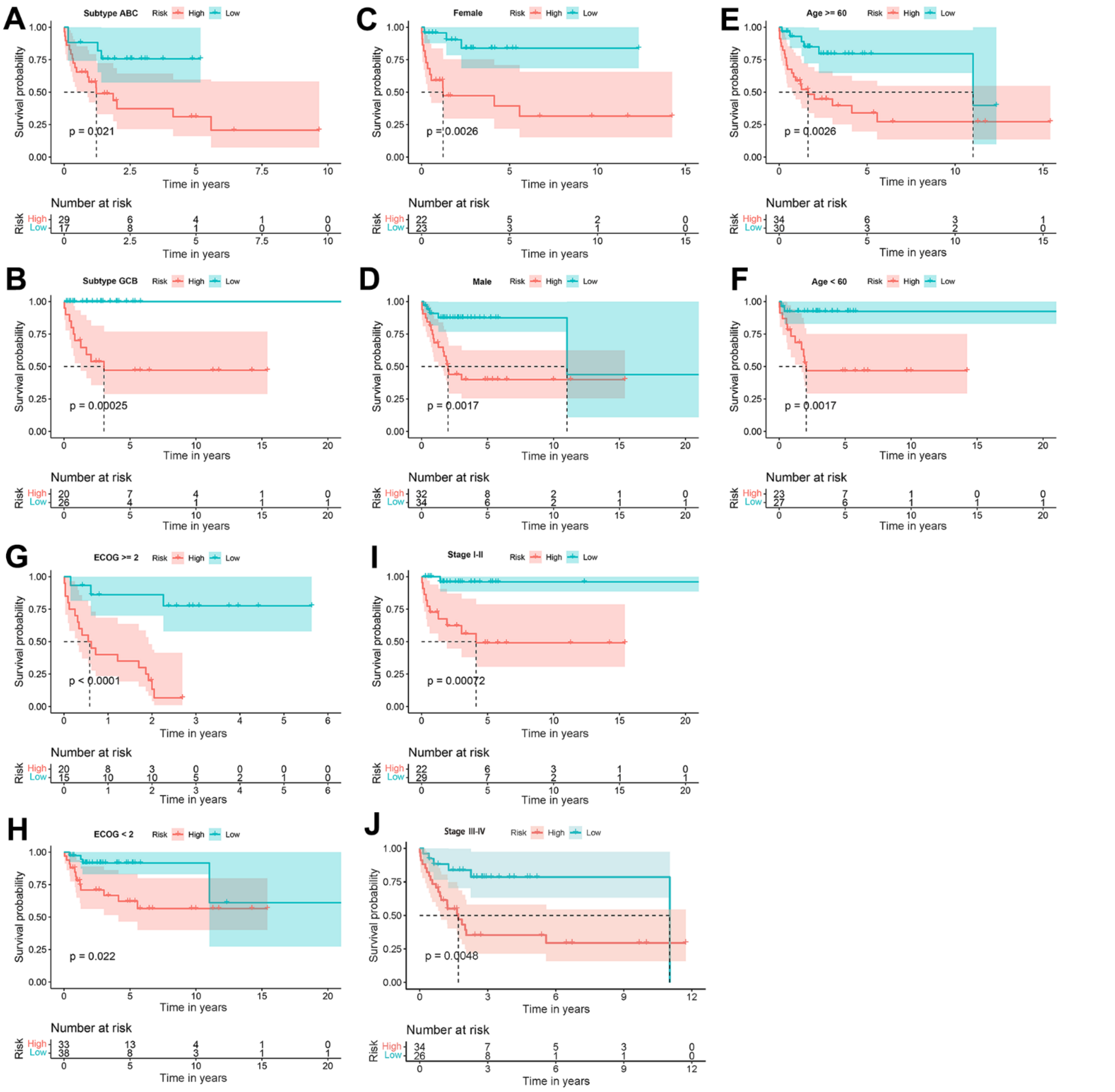

Supplementary Figure 5. Kaplan-Meier curves indicated the survival differences between the low-risk group and high-risk group. DLBCL patients were categorized into different groups according to clinical characteristics including (**A**) subtype ABC, (**B**) subtype GCB, (**C**) female, (**D**) male, (**E**) age >= 60, (**F**) age < 60, (**G**) ECOG >= 2, (**H**) ECOG < 2, (**I**) stage I-II, and (**J**) stage III-IV. Survival comparison of high-risk and low-risk groups among patients with different clinical characteristics.
