# Supplementary material for: Prediction significance of autophagy-related genes in survival probability and drug resistance in diffuse large B-cell lymphoma
Source: Aging (Albany NY). 2024 Jan 17;16(2):1049–76. doi: 10.18632/aging.205282 (PMC10866451; doi:10.18632/aging.205282)
Supplement: Supplementary Table 1 [file aging-16-205282-s002.pdf]

SUPPLEMENTARY TABLE

Supplementary Table 1. A set of 80 genes by overlapping three subsets of autophagy-related genes from HADb, HAMdb, and AUTOPHAGY DATABASE.

Gene symbol
AMBRA1
ATF4
ATG12
ATG16L1
ATG2A
ATG2B
ATG3
ATG4A
ATG4B
ATG4C
ATG4D
ATG5
ATG7
ATG9A
ATG9B
BAG3
BCL2
BCL2L1
BECN1
BNIP3
CALCOCO2
CAPN1
CAPN10
CAPNS1
CDKN1B
CDKN2A
CTSD
EGFR
EIF2AK2
EIF2AK3
EIF2S1
EIF4EBP1
ERN1
FKBP1A
FOXO3
GABARAPL1
GABARAPL2
GOPC
HDAC6
HIF1A
HSPA8
IRGM
ITPR1
LAMP2

MAP1LC3A
MAP1LC3B
MAPK8
MTOR
NBR1
NFE2L2
PARK2
PINK1
PRKAA1
PRKAA2
PRKCD
PRKCQ
PTEN
RAB7A
RB1CC1
RGS19
RHEB
RPS6KB1
RPTOR
SESN2
SH3GLB1
SIRT1
SQSTM1
STK11
TBK1
TMEM74
TP53INP2
TSC1
TSC2
ULK1
ULK2
UVRAG
WDFY3
WIPI1
WIPI2
ZFYVE1
